# Comparative predictive ability of visit-to-visit HbA1c variability measures for microvascular disease risk in type 2 diabetes

**DOI:** 10.1186/s12933-020-01082-9

**Published:** 2020-07-06

**Authors:** Chen-Yi Yang, Pei-Fang Su, Jo-Ying Hung, Huang-Tz Ou, Shihchen Kuo

**Affiliations:** 1 Institute of Clinical Pharmacy and Pharmaceutical Sciences, College of Medicine, National Cheng Kung University, 1 University Road, Tainan, 701 Taiwan; 2 Department of Statistics, National Cheng Kung University, Tainan, Taiwan; 3 Department of Pharmacy, College of Medicine, National Cheng Kung University, Tainan, Taiwan; 4 Department of Pharmacy, National Cheng Kung University Hospital, Tainan, Taiwan; 5 Division of Metabolism, Endocrinology & Diabetes, Department of Internal Medicine, University of Michigan, Ann Arbor, MI USA; 6 Michigan Center for Diabetes Translational Research, University of Michigan, Ann Arbor, MI USA

**Keywords:** HbA1c variability, Microvascular disease, Type 2 diabetes

## Abstract

**Background:**

To assess the associations of various HbA1c measures, including a single baseline HbA1c value, overall mean, yearly updated means, standard deviation (HbA1c-SD), coefficient of variation (HbA1c-CV), and HbA1c variability score (HVS), with microvascular disease (MVD) risk in patients with type 2 diabetes.

**Methods:**

Linked data between National Cheng Kung University Hospital and Taiwan’s National Health Insurance Research Database were utilized to identify the study cohort. The primary outcome was the composite MVD events (retinopathy, nephropathy, or neuropathy) occurring during the study follow-up. Cox model analyses were performed to assess the associations between HbA1c measures and MVD risk, with adjustment for patients’ baseline HbA1c, demographics, comorbidities/complications, and treatments.

**Results:**

In the models without adjustment for baseline HbA1c, all HbA1c variability and mean measures were significantly associated with MVD risk, except HVS. With adjustment for baseline HbA1c, HbA1c-CV had the strongest association with MVD risk. For every unit of increase in HbA1c-CV, the MVD risk significantly increased by 3.42- and 2.81-fold based on the models without and with adjustment for baseline HbA1c, respectively. The associations of HbA1c variability and mean measures with MVD risk in patients with baseline HbA1c < 7.5% (58 mmol/mol) were stronger compared with those in patients with baseline HbA1c ≥ 7.5% (58 mmol/mol).

**Conclusions:**

HbA1c variability, especially HbA1c-CV, can supplement conventional baseline HbA1c measure for explaining MVD risk. HbA1c variability may play a greater role in MVD outcomes among patients with relatively optimal baseline glycemic control compared to those with relatively poor baseline glycemic control.

## Background

Intensive glycemic normalization may not be the only goal to ensure optimal clinical outcomes in people with diabetes [1]. Glucose variability, another indicator of glycemic control, has recently been shown to have an additive or even better predictive effect for diabetic outcomes than a single glycemic level target. Glucose variability comprises (1) glycemic variability, reflecting within-day (daily) or between-day (day-to-day) blood glucose fluctuations [2], and (2) hemoglobin (Hb) A1c variability, reflecting visit-to-visit HbA1c changes over longer periods of time [3]. The biological plausibility of a link between glucose variability and the progression of diabetic vascular complications has been proposed, including active oxidative stress that is associated with not only daily glucose variability (measured by the mean amplitude of glycemic excursions; MAGE) but also day-to-day glucose variability (assessed by the mean of daily differences) [4] and proinflammatory cytokines as a result of hypoglycemia [5].

However, the importance of glucose variability on diabetic outcomes is still under debate because of inconclusive evidence [6, 7]. Shorter-term glycemic variability may not be sufficient to explain chronic diabetic complications. Evidence on the association between HbA1c variability and diabetic outcomes is relatively robust. In patients with type 1 diabetes, HbA1c variability was found as an important risk factor for vascular complications such as cardiovascular diseases (CVDs) [8], microvascular diseases (MVDs) [8–11], and hospitalized hypoglycemia [12]. In patients with type 2 diabetes, although cumulative evidence shows the predictive role of HbA1c variability in the risks of hypoglycemia [12], CVDs [13–20] (such as subclinical left ventricular remodeling and dysfunction [21], reduced baroreflex sensitivity [22], and high thrombotic risk [23]), and all-cause mortality [15–20, 24–30], studies on MVDs [15, 19, 22, 31–37] are relatively limited and yield inconsistent results [34–36].

HbA1c variability can be measured as the following three metrics: (1) intra-individual standard deviation (HbA1c-SD), (2) coefficient of variation of HbA1c (HbA1c-CV), which is a normalized variability measure as the ratio of HbA1c-SD to intra-individual mean (HbA1c-mean), and (3) HbA1c variability score (HVS), which can be interpreted as the percentage of total HbA1c measures that vary by 0.5% (5.5 mmol/mol) [28]. However, there is lack of evidence for examining the comparative predictive abilities of various HbA1c variability measures for diabetic outcomes; most studies considered only one [13–15, 17, 24–28, 31–36] or two [16, 29] variability measures. Although Tseng et al. used three HbA1c variability measures (i.e., HbA1c-SD, HbA1c-CV, and HVS) to assess the role of mean HbA1c in the association between HbA1c variability and all-cause mortality, they did not further explore the comparative predictive abilities among these HbA1c measures [27].

The present study assesses the associations of various HbA1c measures, including three variability measures (HbA1c-SD, HbA1c-CV, and HVS) and two HbA1c mean estimates (overall mean over the study follow-up period and yearly updated means over time), with the risk of composite MVD events while adjusting for the baseline HbA1c level and potential confounding characteristics in a type 2 diabetes population.

## Methods

### Data source

This retrospective cohort study utilized linked individual patient records from the electronic hospital records of NCKUH with the claims data of Taiwan’s National Health Insurance Research Database (NHIRD). NCKUH is a leading medical center in southern Taiwan, with an average of 5800 outpatient visits and 152 inpatient visits per day. The claims data of NHIRD derived from the National Health Insurance (NHI) program, which is a mandatory-enrollment, single-payment system that covers over 99% of Taiwan’s population. Specifically, the data were connected between NCKUH and NHIRD by a deterministic linkage based on individual age, gender, admission date, and diagnosis codes per visit. Data from both NCKUH and NHIRD were used to confirm patients’ demographics, comorbidities, complications, and treatments. Study biomarkers (i.e., HbA1c and hemoglobin) were obtained from NCKUH records.

### Cohort identification

Patients diagnosed with type 2 diabetes (International Classification of Diseases, Ninth Revision, Clinical Modification (ICD-9 CM) codes: 250.x0, 250.x2, x = 0–9) were identified from the NHIRD if they had: (1) at least two outpatient visits with diabetes diagnosis in the year, (2) at least one inpatient visit with diabetes diagnosis, or (3) one outpatient visit with diabetes diagnosis and any prescription of a glucose lowering agent within the same year. Incident cases were defined as those diagnosed with diabetes and without medical history of diabetes for the preceding 3 years. The index date for study patients was defined as the first date of HbA1c examination taken at NCKUH during 2011/1/1 to 2017/5/1. When measuring HbA1c variability, patients without any HbA1c records during the follow-up were excluded. The follow-up period was assessed from the index date until death, loss to follow-up, event occurred, or end of follow-up (2017/5/1), whichever came first.

### Study variables

The primary outcome of interest was composite MVD events that occurred after the index date to the end of follow-up, which included retinopathy, nephropathy, and neuropathy measured using ICD-9-CM disease and procedure codes (detailed codes are presented in Additional file 1: Table S1).

Six HbA1c measures were implemented in this study. First, the baseline HbA1c values at the index date (as a single-point measure, denoted as HbA1c-index) was measured. Second, the HbA1c from the index date to the end of follow-up was measured in terms of three variability measures, namely HbA1c-SD, HbA1c-CV, and HVS, and two mean indices, namely the overall mean of HbA1c in the entire study follow-up period (denoted as HbA1c-mean_overall_) and the annual average of HbA1c values in each follow-up year (HbA1c-mean_yearly_), which referred to the yearly updated mean. HVS was calculated as the number of times HbA1c changed by > 0.5% (5.5 mmol/mol) divided by the total number of HbA1c measurements. For example, if a patient had five successive examinations of HbA1c during the follow-up with values of 6.5% (48 mmol/mol), 7.2% (55 mmol/mol), 7.5% (58 mmol/mol), 7% (53 mmol/mol), and 7.2% (55 mmol/mol), respectively, then the variability score for this patient was calculated as 50% (i.e., 2/4 × 100%).

Other study variables were patient demographics (i.e., age, gender) at the index date, diabetes duration (the time from type 2 diabetes diagnosis to the index date), the results of laboratory tests measured from the index date to the end of follow-up (e.g., blood pressure, lipid profile, and renal function), and histories of comorbidities, diabetic complications, antidiabetic treatments, and CVD-related treatment exposure in the year prior to the index date. All study variables are presented in Table 1.

**Table 1:** Characteristics of overall study patients and those stratified by the status of microvascular disease occurrence during the study follow-up

Variable^a^	All (n = 1705)		Without MVD (n = 533)		With MVD (n = 1172)	
	n or mean	% or SD	n or mean	% or SD	n or mean	% or SD
Males (%)^b^	979	(57.42)	321	(60.23)	658	(56.14)
Age at index date^b^	53.14	(12.23)	49.98	(13.44)	54.57	(11.36)
Diabetes duration^c^	6.45	(3.77)	5.75	(3.86)	6.77	(3.69)
Follow-up (years)	1.99	(2.09)	4.01	(1.97)	1.07	(1.37)
Smoking status	64	(3.75)	20	(3.75)	44	(3.75)
Biomarker^b^						
BMI (kg/m^2^)	25.88	(4.12)	25.68	(4.07)	25.96	(4.14)
Smoking status	64	(3.75)	20	(3.75)	44	(3.75)
Mean HDL (mg/dL)	50.31	(13.45)	51.25	(13.81)	49.90	(13.27)
Mean LDL (mg/dL)	105.94	(25.61)	108.35	(25.62)	104.93	(25.56)
Systolic BP (mmHg)	133.44	(15.61)	130.10	(16.06)	134.60	(15.32)
Total cholesterol (mg/dL)	171.50	(30.79)	173.57	(31.30)	170.60	(30.53)
ACR (mg/g)	234.06	(768.93)	49.34	(211.20)	306.27	(886.91)
Hemoglobin (g/dL)	12.97	(1.86)	13.55	(1.68)	12.77	(1.87)
eGFR (mL/min/1.73 m^2^)	74.08	(19.56)	82.18	(10.57)	70.59	(21.42)
WBC (10^3^/μL)	7.50	(2.39)	7.30	(2.67)	7.57	(2.28)
Comorbidity/complication^d^						
Previous CVD events (%)	380	(22.29)	125	(23.45)	255	(21.76)
Previous MVD events (%)	1348	(79.06)	343	(64.35)	1005	(85.75)
Peripheral vascular disease (%)	50	(2.93)	7	(1.31)	43	(3.67)
Atrial fibrillation (%)	96	(5.63)	21	(3.94)	75	(6.40)
History of amputation (%)	3	(0.18)	2	(0.38)	1	(0.09)
Diabetes and CVD-related medication						
Metformin (%)	1148	(67.33)	359	(67.35)	789	(67.32)
Sulfonylurea (%)	1106	(64.87)	311	(58.35)	795	(67.83)
Meglitinide (%)	113	(6.63)	23	(4.32)	90	(7.68)
Acarbose (%)	172	(10.09)	39	(7.32)	133	(11.35)
TZD (%)	191	(11.20)	46	(8.63)	145	(12.37)
DPP-4i (%)	336	(19.71)	72	(13.51)	264	(22.53)
Insulin (%)	191	(11.20)	59	(11.06)	132	(11.26)
Anticoagulants (%)	21	(1.23)	8	(1.50)	13	(1.11)
Agents acting on the renin-angiotensin system (%)	817	(47.92)	179	(33.58)	638	(54.44)
HbA1c variability and mean measure^b^						
HbA1c-SD (%)	0.84	(0.73)	0.82	(0.65)	0.85	(0.75)
HbA1c-SD (mmol/mol)	9.20	(8.00)	9.00	(7.10)	9.30	(8.20)
HbA1c-CV	0.11	(0.08)	0.10	(0.07)	0.11	(0.08)
HSV	40.63	(32.78)	40.56	(33.80)	40.66	(32.39)
HbA1c-mean_overall_ (%)	7.73	(1.30)	7.72	(1.40)	7.73	(1.26)
HbA1c-mean_overall_ (mmol/mol)	61	(14.20)	61	(15.30)	61	(13.80)
HbA1c-mean_yearly_ (%)						
1st year	7.79	(1.40)	7.75	(1.48)	7.80	(1.37)
2nd year	7.62	(1.35)	7.52	(1.32)	7.66	(1.35)
3rd year	7.62	(1.44)	7.46	(1.27)	7.68	(1.50)
4th year	7.54	(1.29)	7.43	(1.14)	7.58	(1.33)
5th year	7.61	(1.28)	7.50	(1.20)	7.65	(1.31)
HbA1c-mean_yearly_ (mmol/mol)						
1st year	62	(15.30)	61	(16.20)	62	(15.00)
2nd year	60	(14.80)	59	(14.40)	60	(14.80)
3rd year	60	(15.70)	58	(13.90)	60	(16.40)
4th year	59	(14.10)	58	(12.50)	59	(14.50)
5th year	60	(14.00)	58	(13.10)	60	(14.30)
HbA1c-index (%)	8.03	(1.83)	8.02	(1.97)	8.03	(1.77)
HbA1c-index (mmol/mol)	64	(20.00)	64	(21.50)	64	(19.30)

*MVD* microvascular disease, *SD* standard deviation, *BMI* body mass index, *HDL* high-density lipoprotein, *LDL* low-density lipoprotein, *BP* blood pressure, *ACR* albumin-to-creatinine ratio, *eGFR* estimated glomerular filtration rate, *WBC* white blood cell; CVD, cardiovascular disease; TZD, thiazolidinedione; DPP-4i, dipeptidyl peptidase-4 inhibitor; HbA1c, hemoglobin A1c; HbA1c-SD, the standard deviation of HbA1c; HbA1c-CV, the coefficient of variation of HbA1c; HSV, HbA1c variability score; HbA1c-mean_overall_, the mean of HbA1c values from the index date until the end of follow-up, including the index date; HbA1c-mean_yearly_, the annual averages of HbA1c values from each year during follow-up, including the index date; HbA1c-index, the value of the first HbA1c examination at National Cheng Kung University Hospital

^a^All variables were measured in the year prior to the index date (not including the index date), except for age, gender, biomarkers, and HbA1c measurements

^b^Age, gender, and HbA1c-index were determined at the index date whereas HbA1c-SD, HbA1c-CV, HSV, HbA1c-mean_overall_, HbA1c-mean_yearly_, and other laboratory data were estimated during follow-up

^c^Diabetes duration was measured as the time from the first date of type 2 diabetes diagnosis to the index date, which was the first date of HbA1c examination taken at National Cheng Kung University Hospital

^d^There were no patients with the history of blindness, and thus the descriptive result for this variable was not presented

### Statistical analyses

Cox proportional hazard models were utilized to examine the association between HbA1c measures and MVD risk, except for the model that included HbA1c-mean_yearly_ (i.e., yearly updated mean over time), which was a time-dependent Cox model. 12 potential confounders and MVD history were selected as covariates and adjusted in the multivariable Cox models because they were clinically important and statistically significantly associated with MVD risk based on the results of univariate Cox regression analyses (see Additional file 1: Table S2).

The Cox modeling analyses of MVD risk were divided into two parts. The first part evaluated the comparative predictive performances of the HbA1c measures (i.e., baseline HbA1c, mean HbA1c, HbA1c variability) for MVD risk. Specifically, seven Cox models with three HbA1c variability measures (HbA1c-SD, HbA1c-CV, and HVS), two HbA1c mean measures (HbA1c-mean_overall_ and HbA1c-mean_yearly_), HbA1c-mean_yearly_ with HVS, and the baseline HbA1c (HbA1c-index), were performed separately and then compared. All measures were treated as continuous variables. Of note, HbA1c-mean_yearly_ and HVS, were simultaneously included in one model to test whether a model with two types of HbA1c measures (i.e., mean and variability) would yield better prediction and to assess the independence of the predictive effect of the variability measure (HVS) for MVD risk from the mean measure (HbA1c-mean_yearly_). The HVS measure, rather than HbA1c-SD or HbA1c-CV, was chosen for this task because combining the HbA1c-mean and HbA1c-SD is similar to the concept of HbA1c-CV (i.e., the ratio of HbA1c-SD to HbA1c-mean) and the combination of the mean measure and HbA1c-CV might be subject to collinearity. The second part of the analyses assessed whether the associations of HbA1c variability or mean measures with MVD risk were independent of the baseline HbA1c (HbA1c-index). Specifically, HbA1c-index, which was treated as a dichotomous variable based on a cut-off point of 7.5% [i.e., HbA1c-index < 7.5% (58 mmol/mol) and ≥ 7.5% (58 mmol/mol)], was thus adjusted as a covariate in the multivariable Cox models for assessing the associations of HbA1c variability and mean measures with MVD risk.

For the sensitivity analysis, we applied a cut-off point of 8.0% (i.e., HbA1c-index < 8.0% (64 mmol/mol) and ≥ 8.0% (64 mmol/mol)] for HbA1c-index to perform the above-mentioned analytic procedures. This HbA1c cut-off point was considered because it is clinically recognized as the optimal HbA1c level for middle-aged or elderly diabetes patients with complications [38, 39]. Moreover, to assess whether the association between the HbA1c measures and MVD risk varied with the baseline HbA1c level, subgroup analyses based on various cut-offs (i.e., < 7.5% (58 mmol/mol) and ≥ 7.5% (58 mmol/mol) in the primary analyses; < 8.0% (64 mmol/mol) and ≥ 8.0% (64 mmol/mol) in the sensitivity analyses) of the baseline HbA1c (i.e., HbA1c-index) were performed. The results of these Cox models are presented as hazard ratios (HRs) and associated 95% CIs. A two-tailed *p*-value of less than 0.05 was considered statistically significant. The concordance statistics for the modeling analyses are also reported to assess the goodness of model fit among models. Moreover, Youden index [40], which is the sum of sensitivity and specificity minus one, was obtained for all HbA1c measures. Generally, Youden index demonstrates the optimal cut-off from the receiver operating characteristic curve for a binary outcome (e.g., MVD risk in this study). All analyses were performed using R software version 3.6.1.

## Results

Table 1 presents the detailed patients’ characteristics for all study subjects and those stratified by the status of MVD occurrence during study follow-up. Among 1705 of study subjects, 57.5% were male, the mean age was 53.14 years (SD: 12.23 years), the mean HbA1c value at the index date (HbA1c-index) was 8.03% (64 mmol/mol) [SD: 1.83% (20.00 mmol/mol)], the mean HbA1c-SD value was 0.84% (9.20 mmol/mol), and there was a trend of slightly decrease in the yearly mean HbA1c values over a 5-year follow-up. 22% and 79% of patients were diagnosed with CVD and MVD, respectively, at the baseline. The results of biomarkers during study follow-up between the MVD and MVD-free subgroups were generally comparable, except for the albumin-to-creatinine ratio.

Results of the two parts of main analyses for the associations between HbA1c measures and MVD risk are presented in the first two sections of Table 2. In the first part of the analyses (without adjustment for HbA1c-index), seven models with different HbA1c measures were performed separately for the prediction of MVD risk. Among these models, all HbA1c measures were significantly associated with MVD risk, except for HVS. In the second part of analyses (where HbA1c-index was adjusted as a covariate in the model), only two HbA1c variability measures, namely HbA1c-CV and HbA1c-SD, were significantly associated with MVD risk. Among these models, the magnitude of association between HbA1c-CV and MVD risk is greatest compared to those for other HbA1c measures (e.g., HbA1c-SD); for every unit of increase in HbA1c-CV, MVD risk significantly increased by 3.42- and 2.81-fold based on the models without and with adjustment for HbA1c-index, respectively. In the third section of Table 2 for the sensitivity analysis with a different cut-off point for the dichotomous variable of HbA1c-index [i.e., < 8.0% (64 mmol/mol) and ≥ 8.0% (64 mmol/mol)], the results (see Table 2) remained consistent with the above results.

**Table 2:** Association between HbA1c measures and microvascular disease risk with adjustment for patient demographics, comorbidities, antidiabetic treatments, and cardiovascular disease-related treatments

Models	HR (95% CI)	Concordance (95% CI)
1st part of analyses: comparative predictive ability of various HbA1c measures for MVD risk^a^		
Model with HbA1c-SD	1.1371 (1.0596;1.2202)***	0.6712 (0.6546;0.6877)
Model with HbA1c-CV	3.4154 (1.6495;7.0718)***	0.6709 (0.6544;0.6874)
Model with HVS	1.0015 (0.9995;1.0035)	0.6680 (0.6511;0.6850)
Model with HbA1c-mean_overall_	1.0918 (1.0359;1.1507)**	0.6715 (0.6550;0.6879)
Model with HbA1c-mean_yearly_	1.0797 (1.0287;1.1333)**	0.6737 (0.6572;0.6901)
Model with HbA1c-mean_yearly_ + HVS		0.6727 (0.6557;0.6896)
HbA1c-mean_yearly_	1.0830 (1.0204;1.1495)**	
HVS	1.0003 (0.9980;1.0026)	
Model with HbA1c-index (as continuous variable)	1.0649 (1.0290;1.1020)***	0.6741 (0.6579;0.6903)
2nd part of analyses: comparative predictive ability of various HbA1c variability and mean measures for MVD risk, independent of baseline glycemic control [HbA1c-index, as dichotomous variable at a cut-off of 7.5% (58 mmol/mol)]^b^		
HbA1c-SD	1.1153 (1.0312;1.2063)**	0.6734 (0.6569;0.6899)
HbA1c-CV	2.8119 (1.2766;6.1941)*	0.6733 (0.6568;0.6898)
HVS	1.0006 (0.9985;1.0027)	0.6709 (0.6540;0.6878)
HbA1c-mean_overall_	1.0614 (0.9982;1.1287)	0.6727 (0.6563;0.6891)
HbA1c-mean_yearly_	1.0532 (0.9939;1.1160)	0.6740 (0.6576;0.6905)
HbA1c-mean_yearly_ + HVS		0.6731 (0.6562;0.6901)
HbA1c-mean_yearly_	1.0590 (0.9907;1.1320)	
HVS	1.0001 (0.9979;1.0024)	
Sensitivity analyses: comparative predictive ability of various HbA1c variability and mean measures for MVD risk, independent of baseline glycemic control [HbA1c-index, as dichotomous variable at a cut-off of 8.0% (64 mmol/mol)]^b^		
HbA1c-SD	1.1089 (1.0222;1.2030)*	0.6735 (0.6570;0.6901)
HbA1c-CV	2.6340 (1.1665;5.9477)*	0.6735 (0.6569;0.6900)
HVS	1.0004 (0.9983;1.0025)	0.6709 (0.6540;0.6878)
HbA1c-mean_overall_	1.0519 (0.9844;1.1194)	0.6724 (0.6888;0.6560)
HbA1c-mean_yearly_	1.0461 (0.9875;1.1083)	0.6740 (0.6575;0.6905)
HbA1c-mean_yearly_ + HVS		0.6732 (0.6563;0.6902)
HbA1c-mean_yearly_	1.0516 (0.9841;1.1237)	
HVS	1.0000 (0.9977;1.0023)	

*HbA1c* hemoglobin A1c, *HR* hazard ratio, *MVD* microvascular disease, *HbA1c-SD* the standard deviation of HbA1c, *HbA1c-CV* the coefficient of variation of HbA1c, *HSV* HbA1c variability score, *HbA1c-mean*_*overall*_ the mean of HbA1c values from the index date until the end of follow-up, including the index date, *HbA1c-mean*_*yearly*_ the annual averages of HbA1c values from each year during follow-up, including the index date, *HbA1c-index* the first HbA1c record taken at National Cheng Kung University Hospital

* *p* < 0.05, ** *p* < 0.01, *** *p* < 0.001

^a^In the first part of the analyses, the individual Cox models were adjusted for a total of 13 covariates, including patients’ demographics, biomarkers, comorbidities and treatments (which were significantly associated with MVD risk and shown in Supplementary Table 2) and MVD history

^b^In the second part of the analyses, the baseline HbA1c level was treated as one of the covariates adjusted for in the Cox models, in addition to the 13 covariates adjusted for in the first part of the analyses

**Fig. 1:**
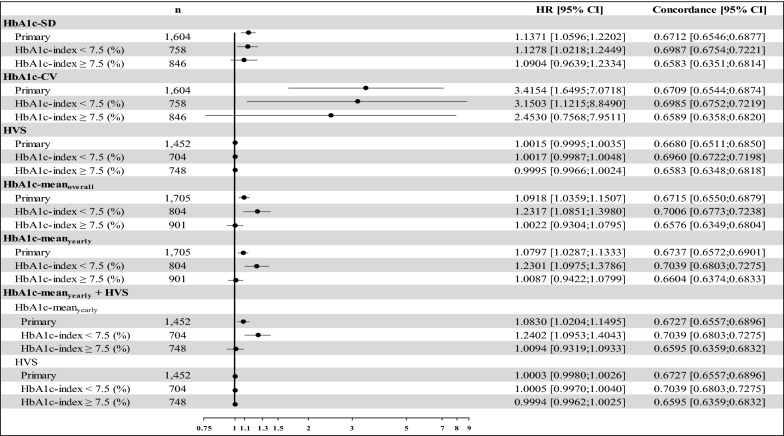
Summary of adjusted hazard ratios for microvascular disease risk by various HbA1c variability and mean measures—primary analysis and subgroup analyses stratified by HbA1c at the index date [i.e., HbA1c < 7.5% (58 mmol/mol) and ≥ 7.5% (58 mmol/mol)]. *HbA1c* hemoglobin A1c, *HR* hazard ratio, *HbA1c-SD* the standard deviation of HbA1c, *HbA1c-CV* the coefficient of variation of HbA1c, *HSV* HbA1c variability score, *HbA1c-meanoverall* the mean of HbA1c values from the index date until the end of follow-up, including the index date, *HbA1c-meanyearly* the annual averages of HbA1c values from each year during follow-up, including the index date, *HbA1c-index* the result of the first HbA1c examination at National Cheng Kung University Hospital

Results of subgroup analyses stratified by the baseline HbA1c level [i.e., HbA1c-index < 7.5% (58 mmol/mol) and ≥ 7.5% (58 mmol/mol)] are summarized in Fig. 1. In general, the associations of HbA1c measures with MVD risk in patients with HbA1c-index < 7.5% (58 mmol/mol) (in terms of statistical significance and the magnitude of association) were stronger compared with those in the patient group with HbA1c-index ≥ 7.5% (58 mmol/mol). A strongest relationship between HbA1c values and MVD risk was found when the HbA1c data were measured as HbA1c-CV; for every unit of HbA1c-CV increase, the risk of developing MVDs significantly increased by 3.15-fold for patients with a baseline level of HbA1c-index < 7.5% (58 mmol/mol) and 2.45-fold for those with a baseline HbA1c-index ≥ 7.5% (58 mmol/mol). Additional file 1: Figure S1 presents the results of subgroup analyses stratified based on a cut-off value of 8.0% (64 mmol/mol) for the HbA1c-index; they are consistent with the results in Fig. 1.

The concordance statistics are reported to indicate the goodness of model fit for the models with different HbA1c measures (Table 2, Fig. 1, Additional file 1: Figure S1). The model fits across the models with different HbA1c measures are considered acceptable (i.e., > 0.65), while the model fit statistics among the subgroup of patients with HbA1c-index < 7.5% (58 mmol/mol) are around 0.7 or above.

Youden index values for all HbA1c measures in the overall study population are summarized in Additional file 1: Table S3. The optimal cut-off of HbA1c-CV suggests 76.29% for developing MVDs (in terms of hazards) at the mean of follow-up of 726 days, with a Youden index value of 0.3576 and corresponding sensitivity and specificity values of 70.88% and 64.88%, respectively.

## Discussion

This is the first study to comprehensively compare the associations of various HbA1c measures, including a single baseline HbA1c value, three HbA1c variability measures, two mean HbA1c estimates, and the combination of the HbA1c variability and mean measures, with MVD risk in a type 2 diabetes population. We found that compared to the single baseline HbA1c value and the mean HbA1c estimates, the HbA1c variability measures generally yielded better predictive performance for MVD risk. HbA1c-CV had the greatest influence on MVD risk, independent of the baseline HbA1c level. The detrimental effect associated with increases in HbA1c-CV in patients with relatively low baseline HbA1c levels (optimal glycemic control) was more apparent than that in those with relatively high baseline HbA1c levels (poor glycemic control).

### Comparison of this study with previous studies on assessing the association between HbA1c variability and MVDs

Several studies have examined the association between HbA1c variability and MVDs in type 2 diabetes patients. Two prospective cohort studies based on 812 and 821 type 2 diabetes patients, over a 4.3-year and a 6.2-year follow-up, from Japan [31] and Taiwan [32], respectively, show that HbA1c-SD is associated with the development of microalbuminuria, independent of the overall follow-up mean HbA1c value. Penno et al.’s study on 8260 type 2 diabetes subjects from the Renal Insufficiency and Cardiovascular Events (RIACE) Italian Multicenter Study [34] reveals the association between HbA1c-SD and renal complications, independent of the overall follow-up mean HbA1c level. However, Penno et al. found that HbA1c-SD had no effects on diabetic retinopathy, which may be mainly dependent on the overall follow-up mean HbA1c level. The cross-sectional study design limits Penno et al.’s study to infer the causality between HbA1c variability and MVD risk. Different from Penno et al.’s findings, two recent studies conducted by Foo et al. [36] and Takao et al. [35] with limited study subjects (398 and 486, respectively) both showed that HbA1c variability measured as HbA1c-SD or HbA1c-CV was predictive for the development of diabetic retinopathy.

Su et al. [33] conducted a 1-year cross-sectional study of 563 type 2 diabetes patients to examine the association between HbA1c variability measured as HbA1c-CV and diabetic peripheral neuropathy. They found that neuropathy events increased with greater HbA1c variability. More recently, a retrospective cohort study conducted by Li et al. [15] analyzed 19,883 type 2 diabetes patients for a range of vascular complications and all-cause mortality, from Tayside and Fife in the Scottish Care Information–Diabetes Collaboration (SCI-DC), and implemented HVS as HbA1c variability. They found the significant predictive role of HbA1c variability for a variety of microvascular complications, including diabetic retinopathy, neuropathy, foot ulcers, and chronic kidney disease.

Our results provide supporting evidence to confirm the important role of HbA1c variability for predicting the risk of MVDs in patients with type 2 diabetes. Unlike previous studies in which only one type of HbA1c variability measure was applied, we employed various HbA1c measures to assess their risk predictive performance for MVDs and reveal the greater impact of increased HbA1c variability measured as HbA1c-CV than other HbA1c measures on MVD risk. In addition, we carefully adjusted for the use of individual antidiabetic treatments and vascular-related medications, which may affect the patients’ risk for developing MVDs. Previous studies either did not adjust for any treatment or medication exposure [31] or did not consider the detailed individual treatments (e.g., only a broader pattern of antidiabetic treatment was measured: non-treatment, oral drugs, oral and injectable drugs, or injectable drugs only [14, 15, 36]). Moreover, baseline glycemic control may be associated with the development and progression of vascular complications. However, most previous studies [15, 31, 32, 34–36] did not explicitly measure and adjust for baseline glycemic control (baseline HbA1c level) in the analyses. We either included the baseline HbA1c level as a covariate in the model or stratified the analyses by the baseline HbA1c value, which minimized the confounding effects from baseline glycemic control. Lastly, considering diverse circumstances in real-world settings, several subgroup and sensitivity analyses were conducted to verify our primary study findings.

### Association of HbA1c-CV with MVD risk in patients with versus those without the relatively optimal baseline glycemic control

We found that the increase in HbA1c-CV had a greater impact on the risk of MVDs among patients with relatively low (optimal) baseline HbA1c levels compared to those with relatively high (poor) baseline HbA1c levels. Statistically, this could be explained by the HbA1c-CV calculation, where the individual HbA1c-SD value is divided by the HbA1c-mean value. Our analyses showed that the HbA1c-mean and HbA1c-SD values in patients with baseline HbA1c < 7.5% (58 mmol/mol) were smaller than those in patients with baseline HbA1c ≥ 7.5% (58 mmol/mol) (Additional file 1: Table S4). This implies that the influence of a one-unit change in HbA1c-SD on the relatively smaller HbA1c-mean value among patients who had the relatively optimal glycemic control at baseline would be greater than that on the relatively larger HbA1c-mean value among those who did not. In addition, owing to a smaller SD for HbA1c-CV among patients with baseline HbA1c < 7.5% (58 mmol/mol), the HbA1c-CV would be more sensitive and the impact of the HbA1c-CV on MVD risk would be more likely to achieve a greater level. This finding could be explained from a clinical aspect. HbA1c data measured as a SD over time among patients with the relatively poor glycemic control at baseline are likely to be more variable than those among patients with the relatively optimal glycemic control at baseline (i.e., a larger HbA1c-SD in patients with poor glycemic control versus those with optimal glycemic control as observed in our data in Additional file 1: Table S4). This could be because if patients have poor glycemic control, they would likely have closer monitoring, intensive antidiabetic therapy, or both, and thus the relatively larger change magnitudes in HbA1c levels (the larger HbA1c-SD) owing to glycemic improvement would be expected. In contrast, low visit-to-visit variations in HbA1c levels among patients with a relatively low HbA1c level at baseline are likely observed because of their optimal glycemic control. Future studies on the value of HbA1c variability for predicting clinical outcomes among a heterogenous diabetic population (e.g., different baseline glycemic levels) are warranted to corroborate our findings. In addition, other measures or markers of glucose variability associated with HbA1c levels, oxidative stress, and MVD outcomes in diabetes patients with relatively optimal glycemic control are deserved for further investigation. For example, postprandial hyperglycemia [41], 1,5-anhydro-d-glucitol as a marker of short-term glycemia control [42], and MAGE assessed by continuous glucose monitoring [4], which have been reported to be associated with oxidative stress in diabetes patients with relatively optimal glycemic control (i.e., less than 7.5–8% of HbA1c), may contribute to the MVD risk in this population.

### Study limitations

Several limitations to this study should be noted. First, the interpretation of our results may be limited to a Taiwanese population with type 2 diabetes under a healthcare setting with a universal health insurance coverage. Because regular glycemic checkups (i.e., HbA1c examination is conducted every 1 to 3 months) are reimbursed in such settings, clinical practice is likely to adhere to clinical guidelines and the HbA1c data of individual patients are thus more complete, which ensures the study data quality. Second, because this is a retrospective cohort study, uncorrected confounding may be possible. Nevertheless, we carefully measured and adjusted for possibly-known confounding factors in the analyses, and used subgroup and sensitivity analyses to verify the robustness of our findings. Third, severe hypoglycemia has been shown to be related to HbA1c variability and was not adjusted in our analyses. However, the number of patients experiencing severe hypoglycemic events at baseline was low (7 cases) and no events occurred during the study follow-up, implying that the potential impact of severe hypoglycemia on our analyses might be negligible. Fourth, we aimed to evaluate the impact of HbA1c measures on the risk of composite MVD events, and did not examine the individual sub-types of MVDs. It would be of interest for future research on different MVD event outcomes. Lastly, we did not explore the possible mechanisms that link HbA1c variability and MVD risk, which deserves future research.

## Conclusion

In conclusion, this real-world cohort study considers a variety of HbA1c measures (a single-point value at baseline, variability estimates, and average estimates) to provide supporting evidence for a greater impact of HbA1c-CV on MVD risk over other HbA1c measures, independent of the effects from the baseline glycemic level, patients’ demographics, comorbidities, complications, and antidiabetic and CVD-related treatments. Our results suggest that HbA1c variability can be an addition to conventional baseline HbA1c levels for explaining MVD risk in patients with type 2 diabetes. HbA1c variability may play a greater role for the risk of MVD among patients with relatively optimal baseline glycemic control. These suggest the importance of closely monitoring HbA1c variability in usual practice and using it as a supporting measure along with a single-point HbA1c value to optimize management of microvascular outcomes.

## Supplementary information

**Additional file 1.** Additional tables and figures.

## Data Availability

The data used in this study is from Taiwan’s NHIRD and NCKUH. For NHIRD, the data are available following application (https://dep.mohw.gov.tw/DOS/lp-2501-113.html) for Taiwanese researchers.

## Abbreviations

CVD: Cardiovascular disease
HbA1c: Hemoglobin A1c
HbA1c-CV: Coefficient of variation of HbA1c
HbA1c-index: The baseline HbA1c values at the index date
HbA1c-mean_overall_: The overall mean of HbA1c in the entire study follow-up period
HbA1c-mean_yearly_: The annual average of HbA1c values in each follow-up year
HbA1c-SD: Standard deviation of HbA1c
HR: Hazard ratio
HVS: HbA1c variability score
ICD-9 CM: International Classification of Diseases, Ninth Revision, Clinical Modification
MAGE: Mean amplitude of glycemic excursions
MVD: Microvascular disease
NCKUH: National Cheng Kung University Hospital
NHI: National Health Insurance
NHIRD: Taiwan’s National Health Insurance Research Database
SCI-DC: Scottish Care Information–Diabetes Collaboration
