# Supplementary material for: Comparative predictive ability of visit-to-visit HbA1c variability measures for microvascular disease risk in type 2 diabetes
Source: Cardiovasc Diabetol. 2020 Jul 6;19:105. doi: 10.1186/s12933-020-01082-9 (PMC7339461; doi:10.1186/s12933-020-01082-9)
Supplement: Supplementary file 1 — Additional file 1. Additional tables and figures. [file 12933_2020_1082_MOESM1_ESM.docx]

**Additional file 1: Table S1: International Classification of Diseases, 9^th^ edition, Clinical Modification (ICD-9-CM) codes used to define composite microvascular disease events**

| **Composite microvascular disease^1-4^** | **ICD-9-CM disease code** | **ICD-9-CM procedure code** | **Data source** |
| --- | --- | --- | --- |
| Retinopathy | 250.5x (x=0-9), 362.01, 362.1, 362.83, 362.53, 362.81, 362.82, 362.02, 361.xx (xx=00-99), 369, 364.0, 364.4, 365, 366, 368, 377, 379.23 | 12.41, 12.73, 14.23, 14.24, 14.25, 14.33, 14.34, 14.35, 14.53, 14.54, 14.55, 16.92, 16.99 | Outpatient, ER, or inpatient |
| Nephropathy | 250.4, 580, 581, 581.81, 582, 583, 584, 585, 586, 587, 588, 593, 403, 404, V56, 791.0, V42.0, V45.1, V13.03 | 38.95, 39.27, 39.42, 39.95, 54.98, 55.4, 55.5, 55.6, 39.95, 54.98, 39.95, 54.98 | Outpatient, ER, or inpatient |
| Neuropathy | 356.9, 250.6, 358.1, 951.0, 51.1, 951.3, 354.0-355.9, 713.5, 357.2, 596.54, 337.0, 337.1, 564.5, 536.3, 458, 729.2, 302.72, 607.84 | -- | Outpatient, ER, or inpatient |

Abbreviations: ER, emergency room.

1. *Am J Manag Care* 2012 Nov;18(11):721-6.
2. *Diabetes Care*. 2018 May;41(5):917-928.
3. *Nutr Metab Cardiovasc Dis*. 2014 Jan;24(1):10-7.
4. *BMJ Open*. 2017 Jun 21;7(6):e015117.

**Additional file 1: Table S2: Results of univariate Cox analyses for covariates which were adjusted for in multivariable Cox models for assessing association of HbA1c measures with microvascular disease risk**

| **Variable^a,b^** | **ICD-9-CM disease or ATC drug codes** | **HR [95% CI]** | ***p*-value** |
| --- | --- | --- | --- |
| Age at index date^c^ | -- | 1.02 [1.01;1.02] | < 0.001 |
| Diabetes duration | -- | 1.06 [1.05;1.08] | < 0.001 |
| Gender | -- | 1.13 [1.01;1.27] | 0.032 |
| ACR | -- | 1.00 [1.00;1.00] | < 0.001 |
| eGFR | -- | 0.97 [0.97;0.98] | < 0.001 |
| Heart failure | 428 | 1.62 [1.19;2.22] | 0.002 |
| IHD | 411, 413, 414 | 1.18 [1.01;1.38] | 0.043 |
| PVD | 250.7, 442.3, 443.81, 443.9, 892.1, 443.9, 444.22, 785.4, 400 or 707.1 | 1.93 [1.43;2.62] | < 0.001 |
| Atrial fibrillation | 427 | 1.48 [1.17;1.86] | 0.001 |
| DPP-4i treatment (baseline) | A10BH | 1.45 [1.27;1.67] | < 0.001 |
| Agents acting on the renin angiotensin system | C09 | 1.58 [1.41;1.77] | < 0.001 |
| DPP-4i treatment (follow-up) | A10BH | 0.88 [0.78;0.99] | 0.027 |

Abbreviations: HbA1c, hemoglobin A1c; HR, hazard ratio; ACR, albumin to creatinine ratio; eGFR, estimated glomerular filtration rate; IHD, ischemic heart disease; PVD, peripheral vascular disease; DPP-4i, dipeptidyl peptidase-4 inhibitor.

Notes:

1. Sources of data included outpatient, emergency room, and inpatient records.
2. Age, gender, and diabetes duration were determined at the index date; ACR, eGFR, and DPP-4i treatment (follow-up) were measured from the index date until the end of follow-up, whereas heart failure, IHD, PVD, atrial fibrillation, and DPP-4i treatment (baseline) were identified at the baseline period, which was one year before the index date.
3. The index date refers to the first date of the hemoglobin A1c (HbA1c) test taken at National Cheng Kung University Hospital.

**Additional file 1: Figure S1: Summary of adjusted hazard ratios for microvascular disease risk by various HbA1c variability and mean measures – primary analysis and subgroup analyses stratified by HbA1c at the index date (i.e., HbA1c < 8.0% [64 mmol/mol] and ≥ 8.0% [64 mmol/mol])**

Abbreviations: HbA1c, hemoglobin A1c; HR, hazard ratio; HbA1c-SD, the standard deviation of HbA1c; HbA1c-CV, the coefficient of variation of HbA1c; HSV, HbA1c variability score; HbA1c-mean_overall_, the mean of HbA1c values from the index date until the end of follow-up, including the index date; HbA1c-mean_yearly_, the annual averages of HbA1c values from each year during follow-up, including the index date; HbA1c-index, the result of the first HbA1c examination at National Cheng Kung University Hospital.

**Additional file 1: Table S3: Results of Youden index with corresponding sensitivities and specificities in the study cohort with a mean of follow-up period of 726 days for prediction models with various HbA1c measures for microvascular disease risk**

| **Model** | **Sensitivity (%)** | **Specificity (%)** | **Youden index** | **MVD risk under the optimal cut-off of HbA1c measures (predicted hazards)** |
| --- | --- | --- | --- | --- |
| HbA1c-index | 76.10 | 61.02 | 0.3712 | 0.7303 |
| HbA1c-SD | 74.45 | 61.84 | 0.3629 | 0.7301 |
| HbA1c-CV | 70.88 | 64.88 | 0.3576 | 0.7629 |
| HVS | 73.18 | 62.07 | 0.3524 | 0.7451 |
| HbA1c-mean_overall_ | 73.15 | 64.09 | 0.3724 | 0.7568 |
| HbA1c-mean_yearly_ | 77.39 | 63.56 | 0.4095 | 0.7382 |
| HbA1c-mean_yearly_ + HVS | 71.16 | 69.79 | 0.4095 | 0.7905 |

Abbreviations: HbA1c, hemoglobin A1c; HbA1c-index, the first time of measuring HbA1c; HbA1c-SD, the standard deviation of HbA1c; HbA1c-CV, the coefficient of variation of HbA1c; HSV, HbA1c variability score; HbA1c-mean_overall_, the mean of HbA1c values from the index date until the end of follow-up, including the index date; HbA1c-mean_yearly_, the annual averages of HbA1c values from each year during follow-up, including the index date, HbA1c-index, the result of the first HbA1c examination at National Cheng Kung University Hospital.

**Additional file 1: Table S4: HbA1c variability stratified by the HbA1c-index of 7.5% (58 mmol/mol) and status of microvascular disease (MVD) occurrence during the study follow-up**

|  | **All population** | | **HbA1c-index < 7.5% (58 mmol/mol)** | | **HbA1c-index ≥ 7.5% (58 mmol/mol)** | |
| --- | --- | --- | --- | --- | --- | --- |
|  | **Without MVD** | **With MVD** | **Without MVD** | **With MVD** | **Without MVD** | **With MVD** |
| HbA1c-SD (%) | 0.8233 | 0.8534 | 0.5694 | 0.6371 | 1.0772 | 1.0381 |
| HbA1c-SD (%) (mmol/mol) | 9.0 | 9.3 | 6.2 | 7.0 | 11.8 | 11.3 |
| HbA1c-mean_overall_ (%) | 7.7203 | 7.7336 | 6.9043 | 6.9792 | 8.5033 | 8.3848 |
| HbA1c-mean_overall_ (mmol/mol) | 61 | 61 | 52 | 53 | 69 | 68 |
| HbA1c-CV | 0.1035 | 0.1064 | 0.0795 | 0.0868 | 0.1276 | 0.1232 |

Abbreviations: HbA1c, hemoglobin A1c; HbA1c-SD, the standard deviation of HbA1c; HbA1c-CV, the coefficient of variation of HbA1c; HbA1c-mean_overall_, the mean of HbA1c values from the index date until the end of follow-up, including the index date; HbA1c-index, the result of the first HbA1c examination at National Cheng Kung University Hospital.
